# Physical Culture, Its Abuse in Foot-ball

**Published:** 1893-12

**Authors:** Robert Newman

**Affiliations:** New York City


### HALL'S
### JOURNAL OF HEALTH

HEALTH—THE POOR MAN'S RICHES, THE RICH MAN'S BLISS.

VOL. XL. DECEMBER, 1893. NO. 12.

### PHYSICAL CULTURE, ITS ABUSE IN FOOT-BALL.

Hurrah for the Blues! more hurrahs for the Orange. Tigers and Bulldogs have departed!

We know now the blessings of a Thanksgiving-day; the city of New York has not been painted red entirely; from the ranks of the well behaved students only fifty-one arrests having been made, and only one captain of a team has been badly hurt. Thank heavens! the excitement is over for this season. The question arises if foot-ball in its present shape is the athletic game to be recommended by a civilized community. There may be an art to make touchdowns, but the perils to limb and life, and the severe injuries and actual deaths in consequence are so great, that there is a serious doubt whether or not such college fun should be encouraged.

Already five deaths have been reported this fall from accidents while playing foot-ball, one each from New Jersey, Indiana, Ohio, Wisconsin and Connecticut. The two last were caused by a fracture or dislocation of a cervical vertebra and crushing of the spinal cord. One of them occurred near Farmington, Conn. The second took place during a game between the elevens of the Toledo (O.) High School and the Adrian (Mich.) College, which was played at Adrian. A Toledo player, Carew, says the report, "had the ball, and downed to save it. Three Toledo boys dropped to save him, and in an instant the Adrian team was upon them. When the struggle was over, Carew remained motionless upon the ground. Upon examination it was found that his body was paralyzed from the chest down, and his spinal column injured, and he died the next morning without having recovered consciousness. Another member of the Toledo team was kicked in the groin, and injured so badly that he could not return to his home, A third player suffered serious injury to his right leg. William Barton, of Norwood, is at the Rhode Island Hospital suffering with a broken leg, the result of a scrimmage in a foot-ball game. In a shanty built on a sunken lot, west of Amsterdam avenue and south of One hundred-and-eighth street is the body of George Diehl, a little fellow ten years old, who died last Saturday. It was said that his death had resulted from injuries that he received while playing foot-ball. English newspapers just received mention the death of a foot-ball player in West Hartlepool, "from the effects of injuries received in a foot-ball match, when he was kicked severely in the stomach."

English medical journals every year report a long list of deaths and casualties from foot-ball. A mortality of five, however, is most startling. The question now arises whether half a dozen deaths and hundreds of serious accidents are not too great a penalty to pay for this sport.

The foot-ball games that occur in this city have degenerated into great professional shows, which are made the excuse for unlimited betting, and which end in vulgar carousals. It is quite time for college authorities to interfere.

The object of this article is to emphasize the necessity of physical culture for every body, but particularly for the adolescence as an educational measure, in order to raise the physical standard of the present and following generations. But brutal and dangerous sports must be eliminated from scientific and healthy exercises. The dangers of the other extreme for our children lurk in modern improvements, luxuries, comforts, present fashionable manners and frivolities, forcing children like hot-house plants, which without physical exercise tend to degenerate the individual and thereby the community.

Formerly children were hardened , and seldom had more diseases than measles and perhaps chicken-pox; now the fashionable mother bundles her offspring up almost to suffocation, is afraid of fresh air or a pleasant refreshing breeze, and the consequence is, that every child is expected to have all children diseases, according to the nomenclature of a cyclopedia. There is great danger in the present system, and it is a duty to give a warning, by raising the signal in large letters: Danger! Stop!

The mind can only be developed, if brain force lives in a healthy frame.

WHAT IS PHYSICAL CULTURE?

By physical education is not only meant the development of muscle, but of the entire physique, or material body; but it does more than this, for it educates the mind and strengthens the morals, because the body and mind are intimately connected, and morals depend largely upon their normal health and vigor.

The objects of physical education must, therefore, be admitted to be a high development of the body, of vigor of mind and moral courage. Ancient Europe practiced physical education to a very large extent ; especially was this true in Greece and Italy.

But what are the effects of exercise ? Exercise increases both the quantity and quality of muscle, causing it to grow larger, firmer and more powerful. Well worth recollecting is the fact that a good muscular condition once attained is not lost.

It is impossible for the muscles to undergo much development without implicating the bones. Because of the way muscles are attached to the bone, the latter, during exercise, receives an increased blood supply, is better nourished and grows. Of course this adds to the weight and increases the stability of the body. Joints even enlarge quite rapidly during a first course of training.

The advantage derived by the nervous system from exercise, is almost universally underestimated.

Again, all this exercise of and improvement in bone muscle and nerve tissue is not without effect upon the viscera, such for instance as the heart, lungs, stomach, liver and kidneys. Each one is developed with the other parts of the body. Proper systematic exercise is curing many dyspeptics every week. Nervous palpitation of the heart is overcome by exercise, therefore, improves the general condition of the entire body. The actions of the heart and lungs are increased ; this causes a better circulation of blood in the different parts of the body and viscera, and thus promotes digestion, and brain and body growth.

The liver is kept active, and bilious attacks are cut short or entirely avoided, as also constipation and the poisonous headaches which are its results.

How absurd is it, to hear nowadays so often, the child is nervous, and then to be dosed with bromides and narcotics ; and how many ladies take little liver pills, stuff themselves with blue mass, calomel, and buy the golden remedies and patent medicines, which are largely advertised on fences and in the air in town and country ; while systematic physical exercise, and a more regular life would prevent all the bilious and sick headaches from which they suffer.

There are sins of commission and omission. To apply this to the systematic practice of physical culture, we find neglect and abuse. A large percentage of the people ignore and neglect their physical education ; while others abuse it. Some may differ and say that our youths are trained up to the highest perfection, that we have athletic clubs en masse, and of every variety; races are carried on in all branches, and prize fights prove how much the culture of civilization has advanced. This brings us to investigate the present status of physical culture, and to see how far it is abused.

While it is admitted that progress has been made in the development of physical education within the last fifteen years ; let us recite some facts from the years 1851 until the present days.

THE ABUSE.

"Thirty Years in the Metropolis," is a continuous tale in one of our Sunday papers, and in those chapters is more related of New York life, than I dare to tell here, but everything I mention will also be found verified in those columns.

At those times were stationary theatres for every day prize fights of the most brutal character, the last of which was in White street, where a former pugilist of note was the master of ceremony, and admired as a dignitary and hero. Rat pits and pits for dog fights were on our fashionable thoroughfares, where our young gentlemen frequented daily. One of the last in existence was corner Broadway and Thirteenth street. The performances in Harry Hill's Houston street hall were innocent pastimes in comparison to the former mentioned establishments, and it is certainly a sign of progress in civilization that all these places have vanished from publicity.

Nevertheless the training in boxing with gloves is practised up to the present days, and I know that gentlemen in so called good standing present their children as a reward with boxing gloves ; prize fights are arranged with gloves and such shows are better attended than Seidl's classic concerts. The daily newspapers describe such disgusting performances in detail, and the participants are pictured as heroes and champions ; and most of our youths look up to John L. as a divine; with a desire to be like him.

Now let us inquire into the work of our young people who mean to do physical exercise, and we will find that their working is either in a wrong direction or a downright abuse.

In early youth, and even in our schools, they have no systematic physical education, and the calesthenics recently practised in some schools is only an apology for the real. The more advanced scholars have no gymnasium, a course in which should be part of the curriculum of a good school. Even the gymnasium at the West Point Academy is very meagerly equipped, and is located in an unhealthy place on the ground floor, lacking the necessary fresh air and ventilation. The best exercise done by the very few is practised at home with dumb bells and club. Later on our college boys everywhere practise foot-ball. And how proud they are of their achievement, the following paragraph will show, which has been clipped from the World, of Tuesday, January 21st, 1889, as follows :—

SOUVENIRS FOR PRINCETON'S ELEVEN.

A number of the Alumni of the Princeton University have had manufactured by Tiffany some beautiful silver match-boxes with an orange and black pennant in enamel, with the word "Champion" across the face, which are to be presented as trophies to the members of the champion foot-ball team at the Princeton dinner, on the night of the 23d inst. That famous game was played here in this city in the presence of thousands of people of good society ; some papers say, there were twenty-five thousand spectators. Under these circumstances, the question arises : Is the game of foot-ball to be recommended as an æsthetic pastime in the domain of physical culture.

I say no, and consider it hurtful to health, dangerous to body and even life, and not elevating the mind and morals. For this denunciation of a beloved game, which may now be called the national college game, I offer the following reasons. The game is raw and rough, it does in no way elevate the mind or body, and appears to the uninitiated spectator like a rough and tumble fight in which the players shout, scream, tumble on the ground and often seriously hurt each other.

The game does not develop physical education, but is dangerous to health, life and limb. This is proven by the following references of actual casualties in 1889.

On Thanksgiving, when the greatest game in the records of foot-ball in America was played, one member was so badly hurt, that he had to be carried off in an ambulance.

Casualties that have happened during the season of 1889, recently, are as follows :—

At Princeton five cases, among one a fracture of the leg;

At Yale, eight cases, one eight weeks on crutches, some disfigured;

At Harvard, three cases;

At Cornell, twenty-one cases;

At Lehigh University, six cases;

At Wesleyan, nine cases;

This makes in six colleges fifty-two injuries known, besides many unknown and slighter injuries during one season.

Dr. Leuf relates injuries in the Team of Pennsylvania University (Medical Record, December 1, 1888), and says "These injuries are very common with us in our games of foot-ball."

The Lancet, of November 23rd, 1888, reports a death at Bootle, in England, following a play at foot-ball. The player who lost his life was thrown violently and sustained an injury to the spinal cord. The index of the Lanet for the first half of 1889, shows that there have been sixteen paragraphs relative to the accidents in this athletic sport. These injuries are almost invariably due to *severe direct violence*, and may affect muscles, bones, periosteum, ligaments and aponeuroses. Some are due to over-action and occur spontaneously, that is, without direct external injury.

Some rowing clubs abuse by their action the benefits of physical culture. The club house is used as a place for loafing, and dissipation and idleness; whole day's excursions on the water are made without any other purpose than to kill time, if not worse, and the wardrobe of members in rowing exercise are so scanty and décolletté, that in most communities, they would be arrested and indicted for indecent exposure.

By no means do I condemn "rowing," on the contrary it is a very healthy exercise, and the above remarks are only referable to some who abuse the rowing.

New York City. ROBERT NEWMAN, M. D.

[TO BE CONTINUED.]

